# Structure and Properties of Co-Cr-Mo Alloy Manufactured by Powder Injection Molding Method

**DOI:** 10.3390/ma14082010

**Published:** 2021-04-16

**Authors:** Grzegorz Matula, Aleksandra Szatkowska, Krzysztof Matus, Błażej Tomiczek, Mirosława Pawlyta

**Affiliations:** Faculty of Mechanical Engineering, Silesian University of Technology, Konarskiego 18a St., 44-100 Gliwice, Poland; aleksandra.szatkowska@polsl.pl (A.S.); krzysztof.matus@polsl.pl (K.M.); blazej.tomiczek@polsl.pl (B.T.); miroslawa.pawlyta@polsl.pl (M.P.)

**Keywords:** biomaterials, powder injection molding (PIM), debinding, sintering, mechanical properties

## Abstract

Cobalt–chromium–molybdenum alloys samples were obtained by the powder injection molding method (PIM). PIM is dedicated to the mass production of components and can manufacture several grades of dental screws, bolts, stabilizers, or implants. As a skeleton component, ethylene–vinyl acetate (EVA copolymer) with a low temperature of processing and softening point was used. The choice of a low-temperature binder made it necessary to use a coarse ceramic powder as a mechanical support of the green sample during sintering. The injection-molded materials were thermally degraded in N_2_ or Ar-5%H_2_ and further sintered in N_2_-5%H_2_ or Ar-5%H_2_ at 1300 or 1350 °C for 30 min. The structure of the obtained samples was characterized by X-ray diffraction and electron microscopy. Mechanical properties, including hardness and three-point bending tests, confirmed that a nitrogen-rich atmosphere significantly increases the bending strength compared to the material manufactured in Ar-5%H_2_. This is due to the precipitation of numerous fine nitrides and intermetallic phases that strengthen the ductile γ-phase matrix.

## 1. Introduction

Cobalt alloys are commonly used in medical applications, mainly due to a very high biotolerance and corrosion resistance in simulated body fluids [1,2,3]. Corrosion resistance and the lack of toxic effects on the human body directly depend on the alloy’s chemical and phase composition. To ensure a precisely defined chemical and phase composition, the appropriate production methods should be selected together with heat treatment parameters. [1]. Due to the manufacturing method of cobalt-based alloys are divided into three groups [3,4,5]:

- Cast alloys,
- Wrought alloys,
- Powder metallurgy alloys.

The cobalt alloys manufactured using the powder metallurgy (PM) method are alternatives to the casted and wrought alloys [1,3,5]. This PM processing ensures a fine-grained microstructure of cobalt alloys, positively affects carbide grain growth inhibition during crystallization, and gives the possibility of shaping cobalt-based alloy’s chemical composition over a wide range. Equally important is that powder metallurgy reduces the base material consumption during component formation, which positively impacts the process economy [6,7,8,9]. However, compared to cast and wrought alloys, the production of these alloys from powders requires more attention to technological conditions, i.e., the powder molding method, gas atmosphere, sintering temperature, and cooling rate. The mentioned variables strictly affect the density and structure of sintered materials and their mechanical properties [10]. Additionally, attention should be paid to selecting an appropriate gas atmosphere during sintering to protect the alloy from oxidation and contamination [10,11,12,13]. Sintered cobalt-based alloys provide the following benefits [6]:

- Possibility of obtaining complex shapes including those that closely imitate the anatomical structure geometry such as mandibular branch and layered bone structures (cortical bone + spongy bone);
- Possibility of obtaining multi-layered structures with desired and variable physical and chemical properties, including those with a different Young modulus and bending strength;
- Depending on the process parameters (i.e., sintering temperature and time), the material porosity can be regulated so that it is possible to obtain implants dedicated to osteointegration with the mechanical properties more similar to human bones than the alloys manufactured with other methods.

Powder injection molding (PIM) is dedicated to the manufacturing of small components with complicated shapes and for mass production. In this way, screws, plates, and several ranges of implants can be produced. The PIM technology has been adopted to metals from the processing of polymer materials for which it was developed. Since the beginning of the 1980s, implementation of PIM in the advanced metal component production for medicine and precise mechanics has been observed. Based on the reviewed literature and our own experience, a number of benefits results from using PIM in metal biomaterial manufacturing were specified [14,15,16]. One of these benefits is eliminating microfractures and residual tensions, which can appear during the machining of cast and wrought alloys. Additionally, PIM technology components are characterized by better mechanical properties, less surface roughness, and precisely specified and homogeneous chemical composition. The PIM method’s undoubted advantage is the high production efficiency and the possibility of obtaining complicated spatial geometries, including openwork elements with controlled porosity, both closed and open. Technological problems of PIM relate to strict control of process parameters at each production stage, i.e., powder and slurry preparation., forming, debinding, and sintering. However, the high cost of the PM devices and tools excludes the possibility of single or small-lot production, and the necessity to degrade the binder makes it almost impossible to manufacture large components [10,11,12,13,17]. The presented work aimed to develop the methodology for CoCrMo-based alloy manufacturing using the powder injection molding method and to characterize the impact of the sintering atmosphere on the manufactured material structure and properties.

## 2. Materials and Methods

The selection of the binder components, slurry preparation method, and processing temperature-dependent viscosity of the slurry and the results of thermogravimetric tests were presented in previous article [18]. For further tests, CoCrMo alloy powder (64% in vol., chemical composition given in Table 1), paraffin wax (PW) (16%), stearic acid (SA) (4%), and ethylene–vinyl acetate (EVA) (16%) binder as a low temperature softened and processed skeleton polymer were selected. The properties of the used powder are presented in Table 2. The PIM process was made at 170 °C by a mini piston injection machine produced by the Zamak-Mercator company with 12,000 bar of maximum pressure. The paraffin was used to reduce the viscosity of the powder–polymer slurry and enable the solvent debinding. To improve the wettability of the powder, the effect of the proportion of stearic acid (SA) was also investigated. Due to low binder viscosity, as high as 64% powder share could be used.

The homogenized slurry was injection molded using a machine with a rectangular beam-shaped cavity at temperature of 40 °C. By selecting the appropriate injection molding conditions, it was possible to produce beam-shaped samples, which is useful for the planned three-point bending test. The beam length and width were 61 and 10 mm, respectively, and thickness was 2 or 5 mm. Samples were deliberately produced with two different thicknesses to investigate the effect of the amount of material on the binder degradation process.

**Figure 1:**
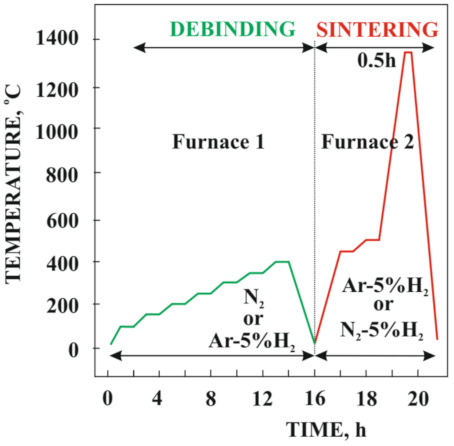
Heat treatment cycle.

The thermal degradation of the binder of injection-molded samples was preceded by a solvent degradation test performed in pure heptane at 25 °C. In general, this solvent process makes thermal debinding easier, with its conditions selected based on the thermogravimetric curve. Both the thermal debinding and sintering were performed in a Czylok pipe oven PRS 75 W (Jastrzębie-Zdrój, Poland) in the N_2_-5% H_2_ and Ar-5% H_2_ atmosphere marked accordingly as an Arcal 15 and Arcal F5 and produced by Air Liquide (Kraków, Poland) [19] with impurity levels as low as 0.02% (H_2_O, O_2_, and N_2_) for Arcal 15 and 0.001% (H_2_O, O_2_) for Arcal F5. The maximum heating rate did not exceed 5 °C/min, while during heating to the temperature of thermal degradation, it was 1 °C/m. The samples were sintered at 1300 °C and 1350 °C (Figure 1).

The beam-shaped samples were subjected to the hydrostatic density test. Microhardness was measured with the Vickers Future-Tech FM-700 (Future-Tech, Milan, Italy) hardness tester with a load of 100 g. Ten measurements were performed for each sample. The three-point bending tests were performed with the appropriate instrumentation used on the Zwick/Roell Z020 (Zwick/Roell, Ulm, Germany) testing machine. The distance between the supporting pins was 25 mm. The microstructure observations were made in a ZEISS SUPRA 35 (Carl Zeiss Instruments, Poznan, Poland) scanning electron microscope (SEM), using the detection of secondary electrons and backscattered electrons at an accelerating voltage of 20 kV and a maximum magnification of 50,000. The S/TEM TITAN 80-300 electron microscope from FEI (Vilnius, Lithuania) with HAADF (High-angle annular dark-field) and BF/DF (Bright field/Dark field) detectors and energy-dispersion spectrometer (EDS) from EDAX were used for investigations of the morphology of nanometric precipitates and its phase identification. The X-ray diffraction (XRD) measurements of the tested materials were performed using the X’Pert PANalitycal (Malvern Panalytical, Malvern, UK) diffractometer with the Bragg–Brentano camera geometry (Kα Co, 2θ range 40 to 120°).

## 3. Results

The use of stearic acid made it possible to reduce the powder–polymer slurry viscosity and enable the insertion of a larger amount of metal powder. In addition, paraffin, although it is not a typical surfactant, decreased the viscosity of the slurry. Moreover, paraffin enables the introduction of solvent initial degradation into the manufacturing process, which facilitates the operation of thermal degradation of the backbone polymer.

**Figure 2:**
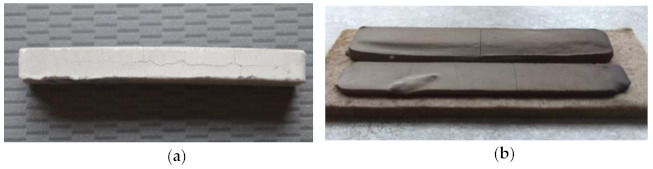
View of sample after solvent debinding in heptane (**a**) and after thermal debinding (**b**).

Paraffin solvent degradation most often takes place in heptane [10,13,18], but in this case, the solvent degradation was causing cracking (Figure 2a). The same effect was obtained by Jiaxin Wen and collaborators for kerosene. The observed cracking was explained by them as result of EVA large volume swelling during solvent debinding [20]. On the basis of this information, the swelling effect of the EVA copolymer in heptane was tested. The pure EVA sample manufactured by injection molding was immersed in heptane for 12 h. After this time, the sample was characterized by high distortion and delamination, which means that heptane should not be used as a solvent in materials containing EVA copolymer as the backbone polymer. Another process problem was the injection-molded sample distortion due to the skeleton polymer’s low softening temperature (Figure 2b).

For this reason, during thermal degradation and sintering, the Co-Cr-Mo samples were embedding into alumina coarse powder. At the temperature needed to densify the injected sample, the surrounding alumina powder remains unsintered, acting as a space filler that mechanically supports the sample during the sintering process. In this way, the injected samples do not deform under their own weight at high temperatures. Additionally, the powder bed acts as a wicking medium to withdraw the molten binder out of the heated part. Therefore, this method of binder degradation can be called a thermal wick debinding process [21]. The results of thermogravimetric tests presented in the article [18] showed that the final debinding temperature of the pure EVA copolymer exceeds 500 °C regardless of whether it takes place in the air or the protective nitrogen atmosphere. The fabricated bending beam samples with EVA as a skeleton polymer subjected to the final debinding at 500 °C in the nitrogen atmosphere showed that the samples turned out to be very brittle and often crack when transferred to the high-temperature furnace to sinter. For this reason, the final temperature of thermal degradation was reduced first to 450 °C and, finally, to 400 °C. Incomplete thermal debinding at 400 °C keeps the skeleton binder residue and residual carbon in their shape and protects the samples from cracking during transport. The isothermal stop was used in the high-temperature oven when heated to the sintering temperature. The final thermal debinding was performed at 500 °C for 1 h to remove the rest of the binder from the material. Subsequently, the samples were directly heated up to the sintering temperature. The results of the density test after sintering at the temperature of 1300 and 1350 °C in N_2_-5%H_2_ are approximately 91 and 94%, and 85 and 92%, respectively, after sintering in Ar-5%H_2_. The low density of materials sintered at 1300 °C/Ar-5%H_2_ that equals to only 85% reduces the strong mechanical properties as shown hereunder. Despite higher material density after sintering in the nitrogen-rich atmosphere, none of sample achieves density close to solid material density. The higher density of the sinters obtained during annealing in the N_2_-5% H_2_ atmosphere can be explained by the diffusion of nitrogen from the furnace atmosphere and the precipitation of nitrides [22,23,24], which was revealed by microscopic examination. In general, the reason for the relatively low density is the porosity of the surface layer confirmed by the scanning electron microscopy, which is presented later in the article. However, this porosity may be beneficial, especially for the durable implants. Drugs or bactericides can be introduced into the open pores to accelerate the healing process of the postoperative wounds. The pores in the top layer appear regardless of the gas atmosphere used during sintering.

**Figure 3:**
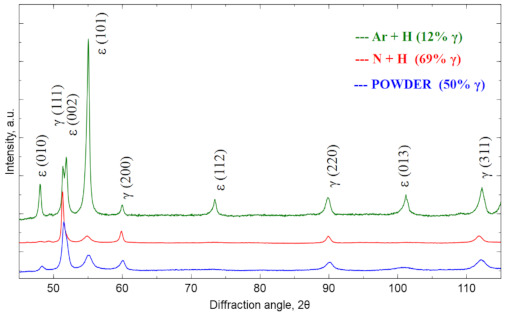
X-ray diffraction pattern for powder and materials sintered at 1350 °C.

Figure 3 shows the X-ray diffractogram for the raw powder and then for the materials sintered in the atmosphere of N_2_-5%H_2_ and Ar-5%H_2_ at 1350 °C. The samples showed the presence of the cubic Co phase marked as γ (COD ID: 96-900-8466, space group Fm-3 m) [25] and the hexagonal Co phase marked as ε (COD ID: 96-900-8492, space group P63/MMC) [25]. The diffraction patterns were normalized to the (200) γ reflex. The relative γ phase fraction was determined based on the intensity analysis of the primary reflections according to the formula Equation (1) [26]:

(1) $$\%\;\gamma \;\mathrm{phase}\;=\frac{I_{\left(200\right)\gamma }\;}{I_{\left(200\right)\gamma }+0.45I_{\left(101\right)\varepsilon }}\times 100$$

where *I*(200)γ and *I*(101)ε are (200)γ and (101)ε intensities. The share of γ fraction is 50% for the raw powder. After sintering in Ar-5%H_2_, the amount of γ fraction is lower than in the raw powder (12%), while after using the N_2_-5%H_2_ atmosphere, the γ fraction is significantly increasing (up to 69%). This large fraction results from the nitrogen-rich atmosphere, which stabilizes the γ-phase during cooling from sintering temperature. Nitrogen-rich cluster formation causes the increase of the energy barrier for transformation from γ to ε, which affects increase of the Co-Cr-Mo alloy elongation [27]. Nitrogen provides also Cr2N precipitates, which are responsible for the increased thermally treated material strength [28]. The sintering conditions were the same in both cases, and the programmed cooling time from 1350 °C to the ambient temperature was 2 h. In fact, when cooling from 600 to 25 °C, this time was even longer due to good oven insulation and its temperature inertia. Thus, the phase composition difference must result only from the changed protection gas mixture used.

**Figure 4:**
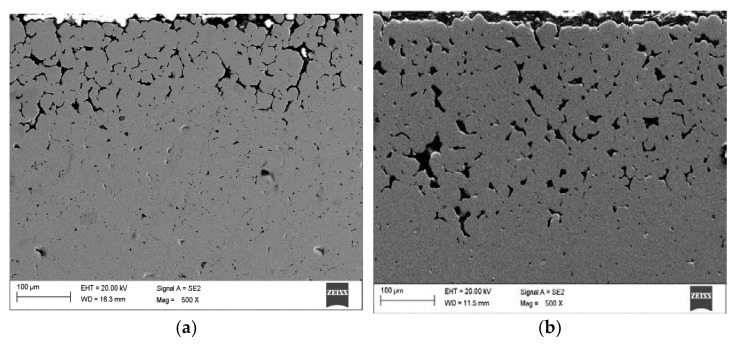
(**a**) Surface layer of sample after debinding under N_2_ and sintering at 1350 °C under N_2_-5%H_2_; (**b**) Surface layer of sample after debinding under Ar-5%H_2_ and sintering at 1350 °C under Ar-5%H_2_.

Figure 4 shows the surface layer of the material that was subjected to thermal debinding in the pure nitrogen atmosphere and then sintering in an atmosphere of N_2_-5%H_2_ at 1350 °C. The top layer is characterized by high porosity. The use of reducing gas mixture used during the thermal debinding and sintering does not ensure sufficient densification of the surface layer. This is due to a high amount of oxides on the CoCrMo powder particle surface as demonstrated by the EDS tests rather than the nitrogen atmosphere during the binder debinding as claimed previously. These oxides are not reduced during thermal debinding and sintering in the hydrogen-rich atmosphere. The binder debinding is undoubtedly most rapid on the top surface. Simultaneously, the residual carbon in the core may initiate sintering, which results in the porosity difference between the core and the top surface. The final thermal debinding temperature was 400 °C, and the samples were then moved to the high-temperature oven, where they were further subject to debinding and sintering in the hydrogen-rich atmosphere. Incomplete thermal debinding protects them against cracking during transport between the heating devices.

**Figure 5:**
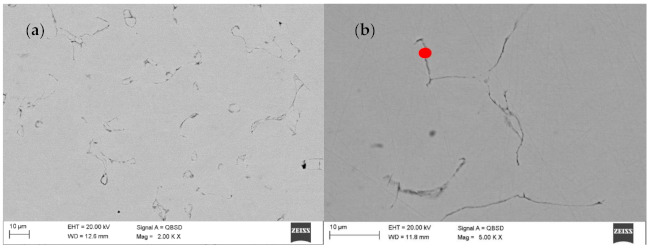
(**a**) Scanning electron microscope (SEM) image of the central part of sample after debinding under Ar-5%H_2_ and sintering at 1350 °C under Ar-5%H_2_; (**b**) SEM image of the central part of sample after debinding under Ar-5%H_2_ and sintering at 1350 °C under Ar-5%H_2_.

**Figure 6:**
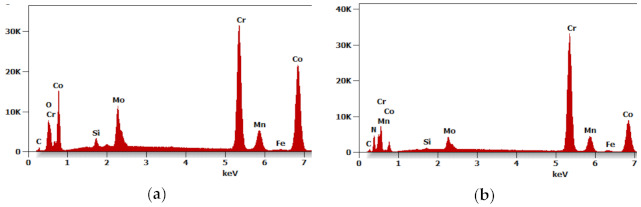
(**a**) Energy-dispersion spectrometer (EDS) analysis from selected area in Figure 10a; (**b**) EDS analysis from selected area.

Further tests of the Ar-5%H_2_ sintered material showed that the sample core has no pores and is dense with multiple oxides often present at the grain boundaries (Figure 5a,b). For example, Figure 6a shows the results of the EDS qualitative analysis. The oxygen content (% at) in the area highlighted in Figure 5b is approximately 18%. This high amount of oxides results from the oxidized batch powder surface *rather* than the process where the hydrogen-rich gas mixture was used within the entire temperature range.

**Figure 7:**
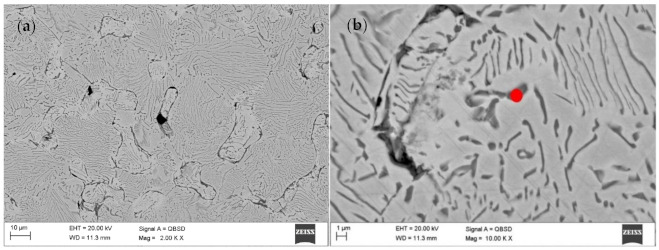
(**a**) SEM image of the central part of sample after debinding under N_2_ and sintering at 1350 °C under N_2_-5%H_2_; (**b**) SEM image of the central part of sample after debinding under N_2_ and sintering at 1350 °C under N_2_-5%H_2_.

Although a reducing atmosphere has been used throughout the heat treatment, a residual carbon content can be expected. At the same time, the hydrogen-rich atmosphere is unable to fully reduce the oxidized surface of the powder during sintering or thermal debinding. The material sintered in N_2_-5%H_2_ contained multiple nitrides (Figure 7a,b). No carbides were found in the produced sinters, suggesting a very low residual carbon level after the binder thermal debinding, and despite the high sintering temperature, no precipitates of these phases occurs. The precipitations showed in SEM images of the N_2_-5%H_2_ sample (Figure 7a,b) were subsequently characterized in detail during the TEM tests. Then, the precipitates seen in the SEM images of the N2-5% H_2_ sample (Figure 7a,b) were characterized in detail during TEM testing. These precipitates occur in the entire volume of the sample and not only at the grain boundaries, which proves the strong diffusion of nitrogen into the sintered particles and are visible in the form of longitudinal and parallel precipitates with comparable distances between them.

**Figure 8:**
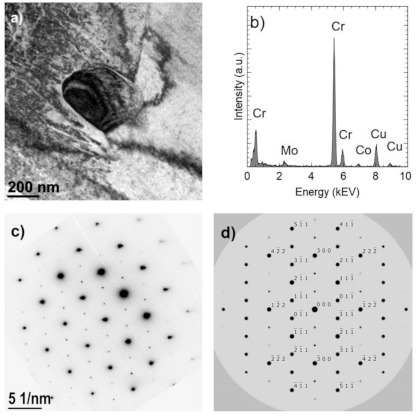
Cr2N precipitate in the sample sintered in N_2_-5%H_2_ at 1350 °C. TEM-BF image (**a**). The result of chemical analysis (Cr 94% at., Co 2% at., Mo 4% at.) (**b**). SAED electron diffraction pattern (**c**). Computer simulation of electron diffraction pattern of Cr2N [011] (**d**).

**Figure 9:**
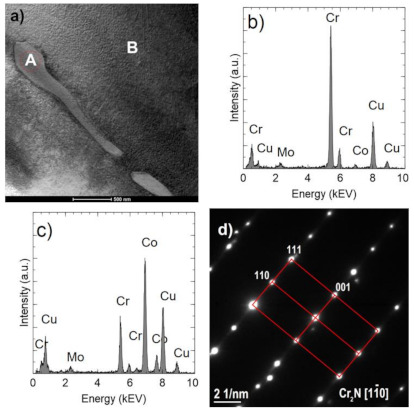
Cr_2_N longitudinal precipitates in the sample sintered in N_2_-5%H_2_ at 1350 °C (**a**). Scheme 96% at., Co 2% at., Mo 2% at.) (**b**). The result of chemical analysis in the area indicated by B (Cr 27% at., Co 70% at., Mo 3% at.) (**c**). SAED electron diffraction pattern for A area (**d**).

Images of precipitates are shown in Figure 8a and Figure 9a, The EDS analyses (Figure 8b and Figure 9b) confirm a high chromium content (approximately 92–96% at.) and a small (less than 6% at.) Co and Mo content. In comparison, the Co fraction in the matrix is approximately 70% at. (Figure 9d). The analyses do not take into account the presence of light elements, including nitrogen. Observed precipitates were identified as Cr_2_N (COD ID: 96-4311894, space group P-3 1 m) based on the selected area electron diffraction (SAED) (Figure 8c and Figure 9d) [29].

**Figure 10:**
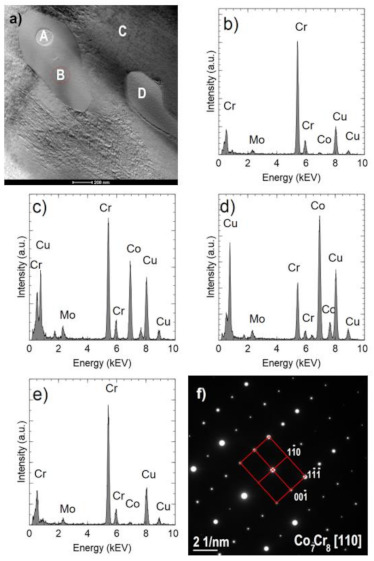
Precipitates in the sample sintered in N_2_-5%H_2_ at 1350 °C. STEM-BF image (**a**). The result of chemical analysis in the area indicated by A (Cr 97% at., Co 1% at., Mo 2% at.) (**b**), in the area indicated by B (Cr 56% at., Co 39% at., Mo 5% at.) (**c**), in the area indicated by C (Cr 29% at., Co 67% at., Mo 4% at.) (**d**), in the area indicated by D (Cr 92% at., Co 2% at., Mo 6% at.) (**e**); SAED electron diffraction pattern obtained for the B area (**f**).

Figure 10a shows an example of an area with two Cr_2_N precipitates. One of them (indicated by A) is surrounded by the σ Co_7_Cr_8_ intermetallic phase, which is marked by B (COD ID: 98-010-2316, space group P42/mnm) [30]. The Co fraction in the σ phase is approximately 39% at. (Figure 10c) and is higher than in the nitride (Figure 10b) but lower than in the matrix (Figure 10d). The σ phase composition was confirmed by the electron diffraction (Figure 10f). Occurrence of the sigma phase surrounding different precipitation or as a binary eutectic has already been demonstrated in earlier studies [31,32]. In this case, the core is chromium nitride, which may arise from the fact that chromium increases the solubility of nitrogen in a cobalt matrix [33]. As already proved [34], the σ phase is metastable; therefore, in the case of carbon-rich cobalt alloys, it turns into M_23_C_6_ carbides at temperatures below 1150 °C. No carbides were found in the analyzed samples, which is another proof of the proper degradation of the polymer binder and a low presence of residual carbon.

**Figure 11:**
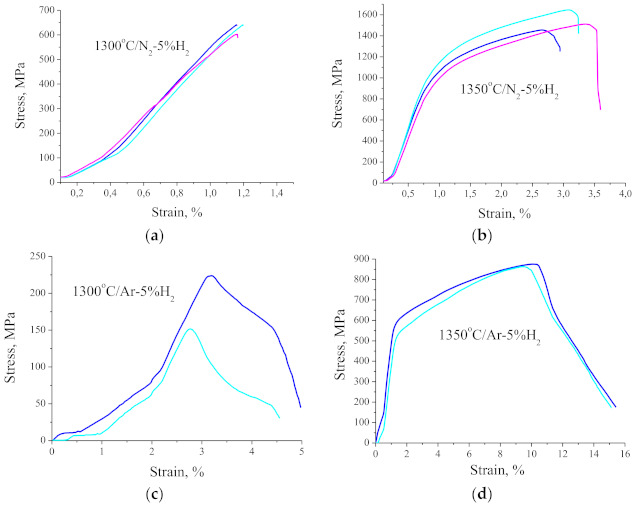
(**a**) Three-point bending tests for samples after debinding under N_2_ and sintering at 1300 °C under N_2_-5%H_2_; (**b**) Three-point bending tests for samples after debinding under N_2_ and sintering at 1350 °C under N_2_-5%H_2_; (**c**) Three-point bending tests for samples after debinding under Ar-5%H_2_ and sintering at 1300 °C under Ar-5%H_2_; (**d**) Three-point bending tests for samples after debinding under Ar-5%H_2_ and sintering at 1350 °C under Ar-5%H_2._

Research on mechanical properties showed that despite numerous oxide precipitations at the grain boundaries, the properties of the sinters are relatively high depending on the temperature and the atmosphere of sintering. The influence of the sintering temperature is of course important not only in terms of the density and thus the porosity of the biomaterials produced but also in terms of the mechanical strength and hardness. The average flexural strength of the sintered beam in N_2_-5% H_2_ at 1300 and 1350 °C is respectively 602 and 1537 MPa, which is definitely higher than the flexural strength of the bone tissue, which is approximately 160 MPa for the femur. The results of those tests are presented in Figure 11a,b. The plastic deformation, i.e., beam deflection is approximately 1% for the materials sintered at 1300 and approximately 3% for the materials sintered at 1350 °C. Moreover, the materials sintered at 1300 °C are brittle and crack due to pores present within the whole material volume. Comparing the test results for sinters formed in N_2_-5% H_2_ and Ar-5% H_2_ at the same temperature, it should be noted that the atmosphere rich in nitrogen makes the sinters much more strengthened while significantly reducing their plasticity. The structural observations confirmed that the materials sintered in the nitrogen-rich atmosphere contain numerous nitrides. The beams sintered at 1350 °C in the Ar-rich atmosphere show approximately 15% deflection, but their bending strength does not exceed 900 MPa (Figure 11d). In comparison, a lower sintering temperature of only 1300 °C results in the maximum bending strength of only 220 MPa while the material retains its plasticity. This material cannot be used due to such low mechanical strength (Figure 11c).

**Figure 12:**
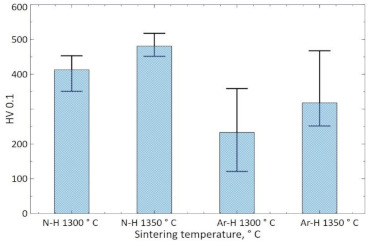
Hardness of samples sintered under N_2_-5%H_2_ or Ar-5%H_2_ at 1300 and 1350 °C.

The hardness of the materials sintered in N_2_-5%H_2_ at 1300 and 1350 °C was 412 and 481 HV0.1 (Figure 12), respectively, with two extreme results rejected and the average value calculated. For the materials sintered in Ar-5%H_2_, this hardness was 232 and 318 HV0.1, respectively. Obtained results confirm that the use of a nitrogen gas mixture during sintering also increases the hardness of the sinters.

## 4. Conclusions

The powder injection molding is the one of the most promising methods for small elements production from powder. Its more common use is limited by the technological difficulties associated with the manufacturing process. In general, according to a report by Grand View Research, Inc. (San Francisco, CA, USA), the global powder injection molding market size is projected to realize USD 6.52 billion by 2025 [35]. For that reason, the applications and development of PIM for implants production that can be additionally modified by sintering atmosphere are very important. The presented article concerns the CoCrMo-based alloy, which has been sintered in an atmosphere enriched with argon or nitrogen after injection molding and debinding, and it describes the improved methodology of manufacturing. The performed analysis confirmed that injection molding at a lower temperature is possible thanks to the use of EVA copolymer, which is additionally economically justified. The thermal debinding of EVA copolymer occurs in the broader temperature range than for the PP or HDPE, which generally facilitates this process [18]. Unfortunately, due to low EVA copolymer softening temperature and material defects such as cracking during paraffin solution debinding in heptane, only thermal wick debinding should be used. The material must be surrounded by the corundum powder acting as a support. A similar solution was described by Manman Zhao et al., who used LDPE as a backbone polymer, because low-density polyethylene has a high swelling ratio in heptane [36]. Using only this type of debinding makes the process longer, taking up to 36 h for the presented bending beams. Higher volume products will require even more time, so the proposed binder composition is more suitable for small or thin-walled components.

Our research confirmed a significant influence of the atmosphere on the structure and properties of the produced material. The use of the Ar-5% H_2_ mixture results in a high proportion of ε phase fraction in relation to γ. No precipitation was observed apart from the oxides present at the grain boundaries in that case. This structure makes the material ductile, and its maximum bending strength does not exceed 900 MPa. Our research confirmed previous reports that nitrogen addition is effective at stabilizing the face-centered cubic (fcc) metallic γ-phase and inhibiting the formation of the σ-phase [24,28]. The share of γ-phase increases more than fivefold. In that case, numerous nanometric precipitates of Cr2N and Co7Cr8 were observed and reliably identified using electron diffraction in conjunction with chemical composition analysis. Mechanical properties, including hardness and three-point bending tests, confirmed that a nitrogen-rich atmosphere significantly increases the bending strength compared to the material manufactured in Ar-5% H2. This indicates that the precipitation of numerous fine nitrides and intermetallic phases strengthens the ductile γ -phase matrix, so its maximum bending strength is approximately 1500 MPa, which unfortunately results in lower plasticity. The same results were shown by Fleming et al. When the nitrogen content increases in the cast CoCrMo alloy, the mechanical properties increase, too, but elongation decreases [37]. Moreover, Tang et al. demonstrate that it is possible to obtain Cr2N phase also during the nitriding because N2 pressure for this precipitation can be lower than for the formation of CrN. The equilibrium pressure for the formation of Cr2O3 is much lower than that for Cr2N or for CrN. For that reason, the mixture of N_2_ with H_2_ was used to prevent the oxidation. In addition, it can be expected that the additional heat treatment of these sinters is to increase their mechanical properties even better.

## Figures and Tables

**Table 1:** Chemical compositions of Co-Cr-Mo alloy powder.

Element	Co	Cr	Mo	Mg	Si	Fe	Ni	W	Al	O2
Mass, %	Bal.	27–30	5–7	<1	<1	<0.75	<0.5	<0.2	<0.1	<0.1

**Table 2:** Properties of Co-Cr-Mo alloy powder.

Shape	Density	Slope Angle Coefficient, Sw	Distribution of Particle Size, μm		
Spherical	8.33 g/cm^3^	2.68	D10 = 15.29	D50 = 30.22	D90 = 52.57

## Data Availability

The data presented in this study are available on request from the corresponding author.
